# Hawthorn Herbal Preparation from *Crataegus oxyacantha* Attenuates In Vivo Carbon Tetrachloride -Induced Hepatic Fibrosis via Modulating Oxidative Stress and Inflammation

**DOI:** 10.3390/antiox9121173

**Published:** 2020-11-24

**Authors:** Alaaeldin Ahmed Hamza, Fawzy Mohamed Lashin, Mona Gamel, Soha Osama Hassanin, Youssef Abdalla, Amr Amin

**Affiliations:** 1 Hormone Evaluation Department, National Organization for Drug Control and Research, Giza 12611, Egypt; fawzy870@hotmail.com (F.M.L.); Monagamil556@yahoo.com (M.G.); 2 Biochemistry Department, Modern University for Technology and information, Cairo 11585, Egypt; phsoha@hotmail.com; 3 Department of Kinesiology, Michigan State University, East Lansing, MI 48823, USA; abdallay@msu.edu; 4 Biological Sciences Collegiate Division, The University of Chicago, Chicago, IL 60637, USA

**Keywords:** liver fibrosis, carbon tetrachloride, hawthorn, antioxidants

## Abstract

Hawthorn (HAW) is a herbal preparation extracted from *Crataegus oxyacantha*. HAW has cardioprotective, antioxidants, anti-inflammatory, and anti-hypotensive effects. HAW’s effect on hepatic fibrosis remains, however, unknown. This study evaluated the impact of HAW on carbon tetrachloride (CCl4)-induced hepatic fibrosis in rats and elucidated its mechanisms. HAW reduced liver index and the serum liver enzyme markers and reduced liver damage, and fibrosis as confirmed by histopathological scoring of hematoxylin-eosin staining. Collagen deposition was reduced in HAW group compared to CCl4 group as confirmed by Masson staining, hydroxyproline content, and both mRNA and protein levels of alpha-smooth muscle actin, collagen 1 and 3. HAW also down regulated the gene expressions of inflammatory markers including interleukin-IL-1β, tumor necrosis factor-α, transforming growth factor-β 1, nuclear factor kappa-B, and cyclooxygenase-2 and decreased the myeloperoxidase activity. The effects of HAW was also associated with decreased levels of hepatic oxidative stress markers (malondialdehyde and P.Carbonyl) and with increased activity of superoxide dismutase. Those effects are possibly mediated by blocking the pro-oxidant machinery and down regulating the inflammatory and profibrotic responses. Finally, chlorogenic acid, epicatechin, rutin, vitexin quercetin, and iso quercetin were identified as the major species of polyphenols of the HAW herbal preparation used here. Therefore, HAW’s potent protecting effects against liver fibrosis predicts a significant beneficial application.

## 1. Introduction

Liver fibrosis and cirrhosis are the main contributors to chronic liver-associated morbidity and mortality [1]. Liver fibrosis results from long standing hepatocellular damage as a normal wound-healing response. Such a response often leads to the production and accumulation of extracellular matrix (ECM) [2] including types 1 and 3 collagens [1,3]. Extended liver injury can be induced by a range of stimuli including viral infection, metabolic disorders, and exposure to drugs and toxic substances [2,4,5]. This chronic liver damage triggers the activation of hepatic macrophages [6] and monocyte-derived macrophages [1,2]. Activation of liver inflammatory cells, in turn, release reactive oxygen species (ROS) [7] in addition to a range of cytokines and other soluble factors. These fibrogenic signals activate hepatic stellate cells (HSC) [2,4,5,8]. Activated HSC then differentiate into myofibroblast-like cells. Those cells are proliferative, contractile, chemotactic, and can synthesize a large amount of ECM including collagen and alpha-smooth muscle actin (α-SMA) [9]. This overproduction of ECM disrupts the normal architecture of the liver and gradually degenerates the normal cellular function of the organ and ultimately causes liver failure [1]. Several cellular mediators of HSC activation and fibrosis in injured/inflamed hepatic tissue include a broad range of fibrogenic and pro-inflammatory factors, such as tumor necrosis factor (TNF-α), nuclear factor kappa-B (NF-κB), cyclooxygenase-2 (COX-2), interleukin-IL-1β and tumor growth factor- β1 (TGFβ1) [1,3,10].

Studies have shown that liver diseases can be avoided and even reversed by natural products working together with silymarin (SIL) [6,11,12,13,14,15]. Use of natural tablets are often preferred due to their low cost, minimum undesirable drug reactions, high safety, and reduced side effects compared to conventional drugs [7]. Silymarin (a main active ingredient of *Silybum marianum*, milk thistle) is a natural drug that is widely used for a variety of health problems including oxidative stress, inflammatory disorders, and liver diseases [16]. Once administered to CCl4-induced model of liver fibrosis, the traditional therapeutic dose [17] of SIL is known to inhibit the fibrogenic mechanism and therefore impedes development of preliminary liver fibrosis [6].

Hawthorn (HAW) is a common name of Crataegus species, a plant of Rosacea family which grows in North Africa, Europe, Asia, and North America [18]. Many HAW species are cultivated for their edible fruit in Asia, Central America, and the Mediterranean region. Traditionally, HAW is used to treat hypertension, angina, arrhythmias, heart failure, anxiety, asthma, dyslipidemia, and indigestion [18,19,20]. Mediterranean HAW species, *Crataegus oxyacanthine* (CO) are cultivated in the Mediterranean and Arab nations [18]. Their leaves, flowers, and fruits are used as herbal medicine specifically for their efficiency treating hyperlipidemia and cardiovascular conditions. Most of HAW’s industrial preparations are also used to treat congestive heart failure [18,19].

CO contains many phytochemicals whose biological activities are useful for promoting human health including rutin, quercitrin chlorogenic acid, hyperoside, vitexin, and caffeic acid [18,19,21]. Thanks to its high antioxidant’s potential and anti-inflammatory effects, CO herbal formulation is currently available and known for its documented effect to prevent or ameliorate the consequences of numerous oxidative stress–related disorders [18,19,21]. Previous investigations have demonstrated that different CO extractions have anti-inflammatory, anti-oxidative stress, and protective effects against tissue damage promoted in animals in association with ischemia–reperfusion [22] and scopolamine [23] in brain, ischemia–reperfusion in heart [24,25], hypercholesterolemia [26], and in high fat food plan [27] in liver. The hepatoprotective effects of standardized extract of HAW was evaluated against acute liver injury induced by CCL4 and hypocholesterolemic agent [26,28].

However, the scientific evidence for supporting hepatic protective effects of CO against liver fibrosis is lacking. Thus, this study was set to evaluate the beneficial effects of the extracts of CO’s (namely of HAW’s) leaves and flowers against CCl4-induced hepatic fibrosis in male rats and to unravel the pathway/s that may mediate such effects.

## 2. Materials and Methods

### 2.1. Chemicals and Reagents

The herbal preparation of hawthorn capsule (Batch No 73995502) was purchased from Nature’s Garden Company (Holland & Barret International Limited, Nuneaton, CV10 7RH, Warwickshire, UK). Each capsule contains 150 mg of hawthorn extracted leaves and flowers of Crataegus oxyacantha. Chloramine T, Folin–Ciocalteu reagent, pyrogallol, superoxide dismutase, catalase (CAT), 2,4-dinitophenylhydrazine, *N*-methyl-2-phenylindol, o-dianisidine, p-dimethyl-amino-benzaldehyde, bovine albumin, and silymarin were purchased from Sigma-Aldrich Chemical Company (St. Louis, MO, USA), rabbit monoclonal anti-α-smooth muscle actin (ab32575) antibody, rabbit polyclonal anti-collagen 1 (ab21287) and 3 antibody (ab7778) were purchased from Abcam and all other chemicals were obtained from local commercial suppliers.

### 2.2. Herbal Drug Preparation

Powder from hawthorn capsules and silymarin were suspended in 5 mL of distilled water before administration. This aqueous extract was then used at a dose of 350 mg/kg body weight which is equivalent to administering a volume of 5 mL/kg body weight.

### 2.3. Animals and Treatments

Twenty-four adult male Wistar albino rats, aged 6 to 8 weeks (200 to 250 g) were supplied by the animal house of the National Organization for Drug Control and Research (NODCAR). Rats were acclimatized for two weeks to the lab conditions. They were housed in polycarbonate clean cages and kept under a 12 h. light/dark cycle at a room temperature of 22–24 °C. They were maintained on standard pellet diet and tap water ad libitum. The protocol was approved and conducted in accordance with standard guide to the care and use of experimental animals (Canadian Council of Animal Care 1993), and was also approved according to the ethical standards by the Institutional Animal Ethics Committee guidelines for animal care and use, NODCAR (NODCAR/II/46/19). Rats were randomly divided into four groups (*n* = 6) and were subjected to the following treatments: The first group (control group) received the vehicle of CCl4 (5 mL corn oil/kg b.wt.) through oral gavage twice a week for 8 weeks and a daily oral dose of water (5 mL/kg b.wt.) for 8 weeks [29,30]. The second group, (CCl4-treated group) received an oral administration of CCl4 (20% CCl4/corn oil; 5 mL/kg body weight twice a week for 8 weeks to produce fibrosis) and a daily oral dose of water (5 mL/kg b.wt) for 8 weeks. The third group (CCl4+ HAW-treated group) received oral administration of CCl4 (20% CCl4/corn oil; 5 mL/kg b.wt.) twice a week for 8 weeks and a daily oral dose of HAW (350 mg/kg b.wt.) for 8 weeks [23,31]. The fourth group (CCl4+ SIL-treated group) was a reference group and received oral administration of CCl4 (20% CCL4/corn oil; 5 mL/kg b.wt. twice a week for 8 weeks) and a daily oral dose of SIL (200 mg/kg b.wt.) for 8 weeks [31,32]. The time interval between CCl4 and each of HAW and SIL was 5 h to avoid the disturbance of absorption of each agent. At the end of the 8th week, all rats were anesthetized with 3% sodium pentobarbital (45 mg/kg, i.p.) and blood samples were collected from the retro-orbital plexus. After the blood was drawn, rats were euthanized by cervical dislocation under diethyl ether anesthesia and the liver was quickly taken out and weighed after being washed with cold normal saline.

### 2.4. Sample Preparation

Livers were collected, weighed, and the liver index was calculated and expressed as liver weight/final body weight. For histopathological examination, parts of collected liver were immediately fixed in 10% buffered formalin. Other parts of livers were rapidly excised and rinsed with ice-cold isotonic saline, then snap-frozen in liquid nitrogen, and stored at −80 °C for later analyses of fibrotic, oxidative stress, and inflammatory markers. To obtain serum, blood was collected in centrifuge tubes and centrifuged in a refrigerated centrifuge (4 °C) at 3000 r.p.m. for 20 min [29,30]. For biochemical determination of the levels of oxidative stress markers, stored parts of livers were homogenized in ice-cold Tris-HCL buffer (150 mM, pH 7.4), of 1:10 wt/v ratio.

### 2.5. Measurement of Serum Biochemical Markers of Liver Damage

Serum levels of aspartate aminotransferase (AST), alanine aminotransferase (ALT), and gamma-glutamyl transferase (GGT) activities as well as albumin, and total bilirubin concentrations were all estimated using Randox (Randox Laboratories Ltd., Antrim, UK) and following their instruction manual.

### 2.6. Histopathological Examination

Liver specimens were fixed in 10% neutral phosphate-buffered formalin and embedded in paraffin before being cut into five-micrometer sections. The hydrated tissue sections were stained with hematoxylin and eosin (H & E) and Masson–Trichrome [33], for the different histological examinations. The sections were examined under an Olympus DX41-light microscope (Honduras St., London, UK). The H & E sections and Masson stain of collagen were graded for average severity of the presence of necrosis, inflammation, fatty metamorphosis, and fibrosis as follows: Grade 0, absent; grade 1, present in one third of the lobules; grade 2, present in two thirds of the lobules; and grade 3, present in all of the lobules [29,30]. In the Masson stain of collagen, digital pictures were taken with identical exposure settings for all sections. Then, positive areas were quantified in ten randomly selected fields (magnification 400×) per individual samples. Area was quantified by using ImageJ software (1.39, NIH-Bethesda, MD, USA) [10].

### 2.7. Immunohistochemically Examination

Sections of paraffin-embedded sections were mounted onto slides, deparaffinized in xylene, and rehydrated in alcohol. The levels of α-SMA and type 1 and 3 collagens were determined by immunohistochemical methods according to the protocols described by Varga [34]. The numbers of α-SMA, were counted in five randomly selected high-power fields (400×) per liver section from each rat. Then, areas of positive immunoreaction of type 1 and 3 collagens were quantified in ten randomly selected fields (magnification 400×) per individual samples by using ImageJ software (1.39, NIH-Bethesda, MD, USA).

### 2.8. Determination of Hepatic Hydroxyproline

Biochemical assessment of hepatic collagen was achieved by measuring hydroxyproline (HP) content in liver samples according to the method of Edwards and Brien [35]. Fifty µl of liver samples or standards were homogenized and hydrolyzed in 0.5 mL 6 N HCl at 110 °C for 18 h. Aliquots of 0.5 mL of 6 N HCl were dried at 60 °C under vacuum. The sediment was dissolved in 400 µL of acetate buffer pH 6.5 then; 0.8 mL of 1.41% Chloramine T reagent dissolved in acetate buffer pH 6.5 was added. After incubation for 25 min at room temperature, 0.8 mL of mixture containing 15 g p-dimethyl-amino-benzaldehyde, and 30% perchloric acid in 60 mL n-propanol was added and the mixture was incubated at 60 °C, for 25 min. After cooling, the absorbance of samples and standards was measured at 550 nm. The results were expressed as µg of HP per milligram of protein.

### 2.9. Oxidative Stress Biomarkers and Myeloperoxidase Activity

The level of malondialdehyde (MDA) in liver tissues was determined as previously described in Gerard–Monnier [36], which is based on its reaction with *N*-methyl-2-phenylindol to form a blue complex with absorption maximum at 586 nm. Two hundred µl of liver sample was added to 650 µL of a solution containing 10 mM *N*-methyl-2-phenylindol in a mixture of acetonitrile/methanol (3:1). The reaction was then started by adding 150 µL of 37% HCl. The 586 nm absorbance was measured upon incubation of reaction mixture at 45 °C for one h. The MDA concentration was determined against a MDA standard curve. The results were expressed as nmol of MDA per milligram of protein.

Hepatic P.Carbonyl contents were determined according to method of Reznick and Packer, [37]. This method is based on spectrophotometric detection of the reaction of 2,4-dinitophenylhydrazine (DNPH) with P.Carbonyl to form protein hydrazones at 370 nm. With cold trichloroacetic acid (TCA, 20 percent final concentration), 400 μL of liver samples are precipitated and then collected for 3–5 min by centrifugation. To give a final protein concentration of 1–2 mg/ml, a solution of 10 mM DNPH in 2 N HCl is added to the protein pellet of each sample, with 2 N HCl only added to the corresponding sample of aliquot reagent blanks. For 1 h with vortexing every 10 min, samples are allowed to stand in the dark at room temperature; they are then precipitated with 10–20 percent TCA (final concentration) and centrifuged for 5 min. The supernatants are discarded, the protein pellets are washed with 10–20 percent TCA again, and then washed three times with 1 mL ethanol/ethyl acetate portions (1:1, *v*/*v*) to remove any free DNPH. The samples are then resuspended with vortex mixing in 6 M guanidine hydrochloride (dissolved in 2 N HCl or 20 mM phosphate buffer, pH 2.3) for 15 min at 37 °C. The results were expressed as nmol of carbonyl group per milligram of protein with molar extinction coefficient of 22,000 M/cm.

The activity of superoxide dismutase (SOD) activity in liver homogenate was determined according to the method described by Nandi and Chatterjee [38]. This method is based on the ability of SOD to inhibit the auto-oxidation of pyrogallol at alkaline pH. In 2 mL of a solution containing 50 mM Tris-cacodylate buffer, pH 8·5, 5 μL of liver sample was applied. The reaction began with the addition of 100 μL of freshly prepared 2·6 mM pyrogallol solution to 10 mM HCl. Absorbance at 420 nm from 1 to 3 min was observed for 2 min. The findings were expressed in tissue homogenate units per mg of protein. One unit of SOD is described as the amount of enzyme required to cause 50% inhibition of pyrogallol auto-oxidation.

Myeloperoxidase (MPO) activity in hepatic homogenate was determined following previously published protocol [39]. Briefly, 100 μL of supernatant was mixed with 2.9 mL of 50 mmol/L potassium phosphate buffer, pH 6.0, containing 0.167 mg/mL o-dianisidine dihydrochloride and 0.0005% hydrogen peroxide. The change in absorbance at 460 nm was measured with a spectrophotometer. One unit of MPO activity is defined as that which degrades 1 μmol of peroxide per minute at 25 °C. The total protein content of heart was determined according to the Lowry method as modified by Peterson [40]. Absorbance was recorded using a PerkinElmer, Lambda 25 UV/VIS spectrophotometer for all measurements.

### 2.10. Quantitative Real-Time Reverse-Transcriptase Polymerase Chain Reaction Analysis

Total RNA was isolated from liver tissue using an RNeasy Mini Kit (QIAGEN, Valencia, CA, USA) and assessed with a dual spectrophotometer Gene JET Kit (Thermo Fisher Scientific Inc., Bremen, Germany, #K0732). Reverse-transcriptase-polymerase chain reaction (RT-PCR) was used for quantitative analysis of gene expression of interleukin-IL-1β, TNF-α, NF-kB, COX2, transforming growth factor-β (TGF-β), α-SMA, Collagen 1, and Collagen 3. The PCR reaction was carried out in 48 well plate Step One real-time PCR systems (Applied Biosystems, Foster city, CA, USA) and results were analyzed using Applied Biosystems software version 2. One microgram of purified RNA from each sample was utilized for reverse transcription with subsequent quantitative PCR amplification with SYBR Hi-ROX Sensi FAST One step kit, catalog number PI-50217V (Bioline, London, UK), in accordance with the kit manufacture’s protocol. The sequence primers for all studied of genes were selected from previous studies and confirmed from GenBank (Table 1). Thermal profile was as follows: 45 °C for 20 min in one cycle (for cDNA synthesis) followed by 10 min at 95 °C for reverse transcriptase enzyme inactivation. Forty cycles of PCR amplification were further carried out as: 10 sec at 95 °C, 30 sec at 58 °C, and 1 min at 72 °C. Changes in the expression of each target gene were normalized relative to the mean cycle threshold values of the housekeeping gene glyceraldehyde 3-phosphate dehydrogenase (GAPDH) by ∆Ct method. All samples were analyzed in triplicate.

### 2.11. Determination of Total Phenolic Content of HAW

The Folin–Ciocalteu reagent was employed to determine the total phenolic content by the by the method of Singleton [41]. Then, 100 μL of Folin–Ciocalteu reagent and 200 μL of sodium bicarbonate solution (10 percent) were applied to 200 μL of each sample solution (2 mg/mL). The mixture was then left at room temperature for 30 min and the absorbance was measured using the UV/Vis Spectrophotometer at 765 nm. In order to map a calibration, solutions with differing gallic acid concentrations were prepared. Results were calculated based on the curve and expressed in milligrams of gallic acid equivalent per g dry weight of crude plant material.

### 2.12. Determination of Total Antioxidant Capacity of HAW

The total antioxidant capacity (TAC) in crude extract was evaluated using ferric reducing antioxidant power (FRAP) assay. The FRAP assay was determined according to the method described by Benzie [42]. The FRAP reagent is composed of a 300 mM acetate buffer pH 3.6, 20 mMFeCl3.6H2O and 10 mM 2,4,6-tripyridyl-striazine solution TPTZ). Then, 200 μL of plant extracts were mixed to FRAP reagent, permitted to stand and absorb for six minutes, 593 nm was noted. The FRAP assay measures the change in absorbance at 593 nm due to the formation of a blue colored ferrous- tripyridyltriazine complex from colorless oxidized ferric form by the action of electron donating antioxidants. Ascorbic acid was used as a standard for the calibration curve.

### 2.13. High-Performance Liquid Chromatography (HPLC) Analysis of Phenolic Compounds of HAW

Powdered samples of HAW (0.5 g) were extracted by ultrasound (for 30 min at 25 °C) using methanol/water (80%, *v*/*v*) and then filtered. Phenolic compounds of HAW extract were analyzed by HPLC following previously reported methods [43]. Briefly, 20 µL of HAW extract was injected onto a reverse phase C18 thermo column (250 mm by 4.6 mm i.d. 4 µm) in Waters 2690 Alliance HPLC (Milford, MA, USA), equipped with a Waters 996 photodiode array detector. Separation and quantification were achieved at 25 °C using gradient conditions. The following gradient of solvents was employed using two solvents: A was formic acid in water, PH = 2.9 and B was acetonitrile/methanol (80:20, *v*/*v*). The gradient program was as the follow: 0–5 min, 10% B; 5–15 min, 10–18% B; 15–25 min, 18% B; 25–30 min, 18–25% B; 30–35 min, 25% B; 35–40 min, 25–35% B; 40–45 min, 35–60% B; 45–50 min 60–10% B; and 50–55 min with 10% B. The flow rate was 1 mL/min and the elution peaks were monitored at 280 nm. The standards of chlorogenic acid, rutin, quercetin, isoquercitrin, epicatechin, and vitexin, were prepared in methanol in the concentration range of 0.01–0.3 mg/mL and were injected in the same chromatographic conditions. The identification of the compounds was based on the comparison of actual retention time of the samples with their respective standards.

### 2.14. Statistical Analysis

All data was statistical analyzed by the SPSS (version 20) statistical program (SPSS Inc., Chicago, IL, USA). The data was expressed as means ± SEM. Statistical significance between treatment groups was carried out by using one-way analysis of variance (ANOVA) followed by Dunnett’s t test-post-hoc analysis test for multiple comparisons with *p* < 0.05 being considered as statistically significant. Figures were done using GraphPad Prism program (version 5) (San Diego, CA, USA).

## 3. Results

### 3.1. HAW Attenuated Liver Lesions in CCl4-Induced Liver Damage

**Figure 1:**
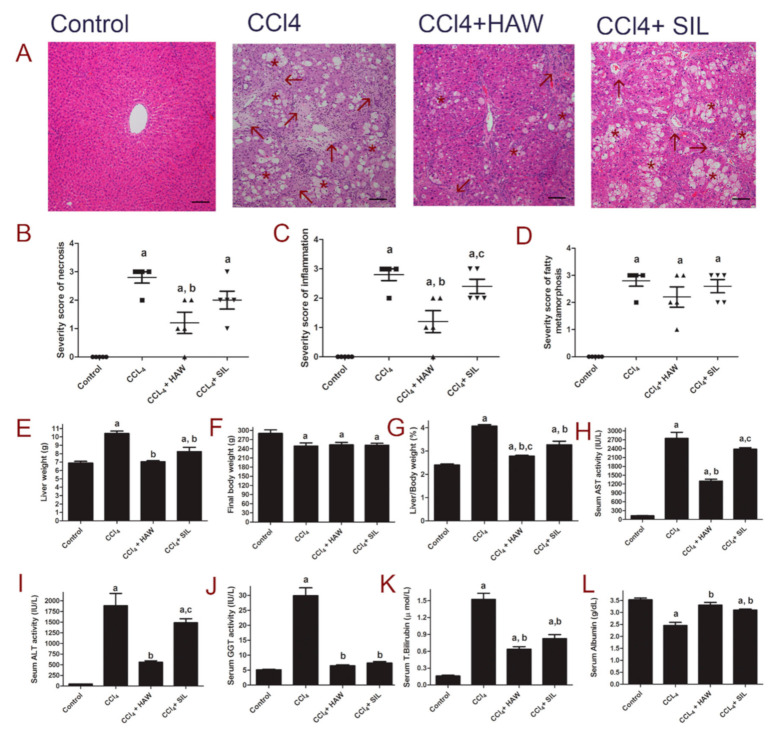
Effects of Hawthorn (HAW) on CCl4-induced liver damage in rats. (**A**) representative images of hematoxylin and eosin (H & E)-stained hepatic sections of the different groups (100×), Scale bar equals 100 µm. Histopathological evidence of CCl4-induced liver damage in rats showing area of necrosis with inflammatory cell infiltration (arrow), and diffuse fatty change (star), (**B**) severity score of necrosis, (**C**) inflammation, and (**D**) fatty metamorphosis. (**E**) liver weight, (**F**) body weight, and (**G**) liver/body weight ratio. Serum markers of liver damage aspartate aminotransferase (AST) (**H**), alanine aminotransferase (ALT) (**I**), gamma-glutamyl transferase (GGT) (**J**) activities and total bilirubin (**K**), and albumin (**L**) levels. Data are presented as means ± SEM. (*n* = 6 per group). a is *p* < 0.05 vs. control group, b is *p* < 0.05 vs. CCl4 group, and c is *p* < 0.05 vs. HAW group.

The histopathological changes in the liver tissues were examined in H & E-stained sections. Evident changes were observed in liver tissues of CCl4-treated group including necrosis, and inflammatory cells infiltration and diffuse fatty changes (Figure 1A,B). Treatment with HAW markedly improved histological scores of necrosis (B), inflammation (C), and fatty degeneration (D) in comparison with CCl4-treated group. Treatment with SIL markedly lowered liver necrosis and inflammatory cell infiltrations. In the CCl4-intoxicated group, liver weight (E), liver/body weight ratio (G), serum aspartate aminotransferase (H), alanine aminotransferase (I), gamma-glutamyl transferase (J), and total bilirubin (K) were markedly increased compared to normal control group. In contrast, treatment with either HAW or SIL markedly reduced both liver and serum markers of hepatotoxicity compared to CCl4-treated group. Furthermore, serum albumin levels (L) significantly lowered in CCl4-treated group, compared to control group. In contrast, treatment with either HAW or SIL markedly raised serum albumin level. Compared to SIL-treated group, rats treated with HAW revealed the best effects in terms of necrosis and fibrosis scores and for serum AST, ALT activities, and for serum albumin levels.

### 3.2. HAW Ameliorated CCl4-Induced Liver Fibrosis in Rats

**Figure 2:**
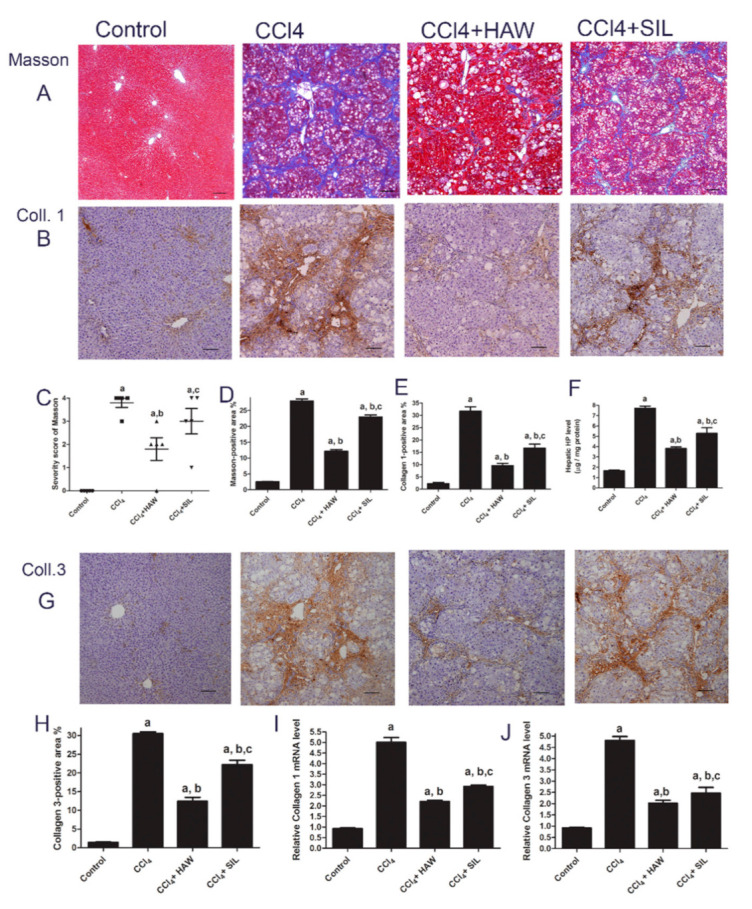
Effects of HAW on the CCl4-induced liver fibrosis rat model. (**A**) representative images of Masson staining (blue area) staining, 100× magnification, scale bar equals 200 µm. (**B**) Representative images of immunohistochemically expression of collagen-1 (brown area). Scale bar equals 100 µm, (**C**) severity score of Masson, and (**D**) Masson-positive area expression as a percentage of the total area (*n* = 5 per group) using the free software NIH ImageJ. (**E**) collagen-1 expression area as a percentage of the total area (*n* = 6 per group). (**F**) hydroxyproline (HP) level in the liver. (**G**) representative images of immunohistochemically expression of collagen-3 in the liver (brown area) scale bar equals 100 µm. (**H**) collagen-3 expression area as a percentage of the total area (*n* = 3 per group). (**I**,**J**) Analysis of type 1 and 3 collagen mRNA expression in the liver by quantitative RT-PCR (*n* = 3 per group). Results were normalized to glyceraldehyde 3-phosphate dehydrogenase (GAPDH) mRNA. Data are presented as means ± SEM (*n* = 6 in each group). a is *p* < 0.05 vs. control group, b is *p* < 0.05 vs. CCl4 group, and c is *p* < 0.05 vs. HAW group.

CCl4-treated group showed significant collagen accumulation by Masson stain as shown in Figure 2A, with deposition of connective tissue and formation of thin septa between hepatic lobules (Figure 2A). The administration of HAW clearly reduced fibrotic septa (Figure 2A) and severity score of Masson and the Masson positive area (Figure 2C,D). On the other hand, treatment with SIL induce some protective effect against collagen deposition. Similarly, measurement of hepatic HP indicates collagen production (Figure 2F). HP content was significantly higher in CCl4-treated group as compared to the control group. HP content was reduced by either HAW or SIL in rats with liver fibrosis. Collagen types 1 and 3 were stained brown when immunohistochemically labeled (Figure 2B,G). These findings were supported by quantification of collagen-1 and 3 immuno-positive areas. Control livers showed low collagen deposition around the blood vessel wall. CCl4 showed a significant deposition of collagen types 1 and 3 as fibrous septa surrounding the lobules (Figure 2B,G). Treatment with HAW decreased the increase in collagen deposition of collagen types 1 and 3 (Figure 2D,G). Furthermore, Figure 2 showed that both of immune staining area (Figure 2E,H) and mRNA expression levels of collagen-1 and collagen-3 (Figure 2H,I) were markedly increased in livers of CCl4- treated rats when compared to controls. However, HAW treatment markedly attenuated both immune staining area (Figure 2E,H) and mRNA expression levels of collagen-1 and collagen-3 (Figure 2I,J) in liver, when compared to CCl4- treated rats. Treatment with HAW showed the best effects on all liver fibrosis markers when compared to SIL-treated group.

### 3.3. HAW Ameliorated Overexpression of α-SMA in CCl4-Induced Liver Fibrosis in Rats

**Figure 3:**
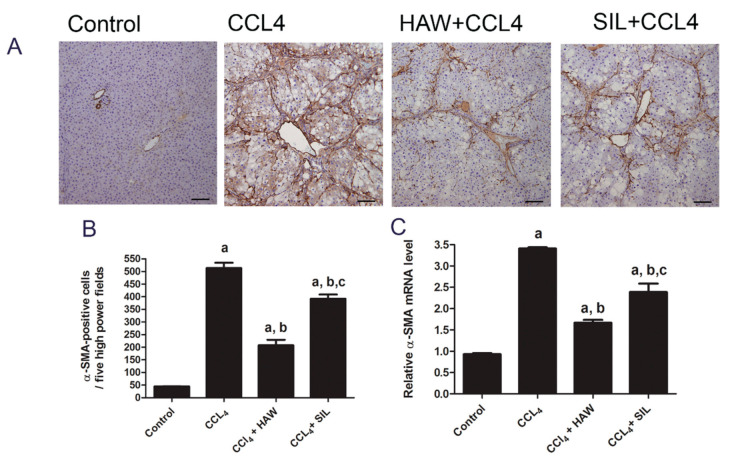
Effects of HAW on the CCl4-induced hepatic stellate cells (HSC) activation in liver fibrosis model. HAW inhibited HSC activation in liver fibrosis. (**A**) representative images of immunohistochemically expression of α-SMA in the liver was determined by immunohistochemistry (brown area), 100× magnification, scale bar equals 100µm. In CCL4 section marked staining for alpha-smooth muscle actin (α-SMA) is found along with the fibrous septa. (**B**) the expression of α-SMA cells in each section was calculated by counting the number of brown staining, α-SMA-positive cells in five fields per section at 400× magnification, *n* = 6 per group). (**C**) the mRNA expression levels of α-SMA were detected by q-RTPCR. Data are presented as means ± SEM, *n* = 3 per group. a is *p* < 0.05 vs. control group, b is *p* < 0.05 vs. CCl4 group, and c is *p* < 0.05 vs. HAW group.

The α-SMA was immunohistochemically assessed as a marker of HSC activation. Positive immune staining of α-SMA was mostly in walls of blood vessels of normal group. In CCL4-treated group, positive immune staining of α-SMA was also located in fibrous septa and areas of fibrosis (Figure 3A,B) which indicating HSC activation into myofibroblasts. In contrast, treatment with HAW markedly (*p* < 0.05) decreased the number of α-SMA positive cells when compared to CCL4-treated group indicating inhibition of α-SMA–expressing myofibroblasts. SIL-treated rats exhibited similar recovery pattern. HAW and SIL significantly decreased the mRNA expression level of α-SMA in rats when compared to those of the CCL4-treated rats (Figure 3C). HAW showed the best effects in of α-SMA inhibition compared to SIL-treated group.

### 3.4. HAW Ameliorated Overexpression of Inflammatory Markers in CCl4-Induced Liver Fibrosis in Rats

**Figure 4:**
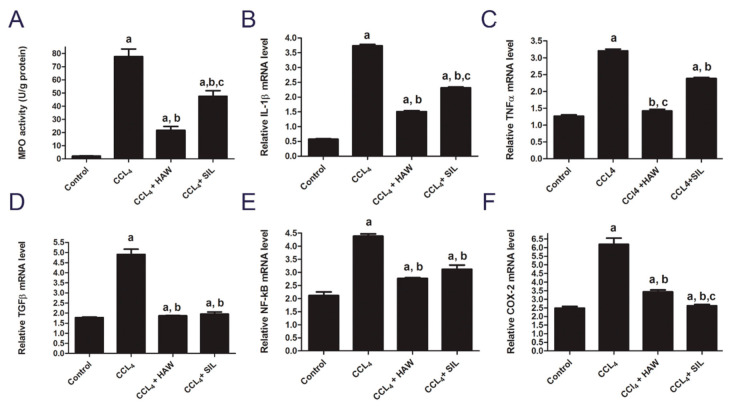
HAW inhibited the overexpression of inflammatory factors in rats. (**A**) myeloperoxidase (MPO) activity, (*n* = 6 per group) and mRNA expression of (**B**) IL-1β, (**C**) tumor necrosis factor (TNF-α), (**D**) TGF β, (**E**) nuclear factor kappa-B (NF-kB), and (**F**) cyclooxygenase-2 (COX-2) in liver tissues (*n* = 3 per group). Results of mRNA expression were normalized to GAPDH mRNA. Data are presented as means ± SEM). a is *p* < 0.05 vs. control group, b is *p* < 0.05 vs. CCl4 group, and c is *p* < 0.05 vs. HAW group.

Figure 4 showed the activity of MPO and the mRNA levels of IL-1β, TNF-α, TGF β, NF-kB, and COX-2 in rat livers. Hepatic MPO activity was carried out as a marker of oxidative stress, inflammation, and tissue neutrophil accumulation and activation (Hillegass et al., 1990). CCl4-treated group induced significant elevation in MPO activity, compared to control group. Treatment with HAW and SIL significantly decreased MPO activity compared to CCl4-treated group (Figure 4). The mRNA levels of IL-1β, TNF-α, TGF β, NF-kB, and COX-2 were significantly (*p* < 0.05) upregulated in livers of CCl4- treated rats compared to control group. Meanwhile, the mRNA levels of IL-1β, TNF-α, TGF β, NF-kB, and COX-2 were significantly downregulated in HAW and SIL groups when compared to control group (Figure 4). Compared to SIL-treated group, inhibition of all the CCl4-induced inflammatory responses was far better with HAW treatment.

### 3.5. HAW Ameliorated Oxidative Stress in CCl4-Induced Liver Fibrosis in Rats

**Figure 5:**
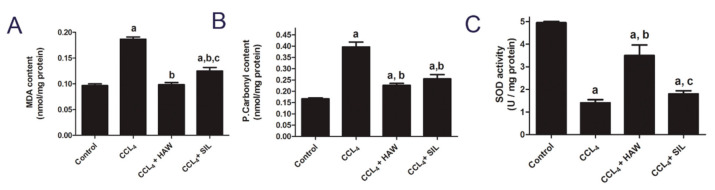
HAW inhibited oxidative stress markers in rats. (**A**) malondialdehyde (MDA) content (**B**) P.Carbonyl content and (**C**) superoxide dismutase (SOD) activity in liver tissues. Data are presented as means ± SEM (*n* = 6 in each group). a is *p* < 0.05 vs. control group, b is *p* < 0.05 vs. CCl4 group, and c is *p* < 0.05 vs. HAW group.

Figure 5 presents the effects of HAW on CCl4-induced oxidative stress damage in rats. There were significant increases in MDA and P.Carbonyl contents and an overall decrease in SOD activity in livers of CCl4-treated group, compared to control group (Figure 5). HAW administration significantly attenuated the increase in hepatic MDA and P.Carbonyl and the decrease in SOD activity, compared to CCl4-treated group. Compared with SIL-CCl4 group, the treatment with HAW proved to be more potent.

### 3.6. Total Antioxidant Capacity and Phenolic Content of HAW

**Figure 6:**
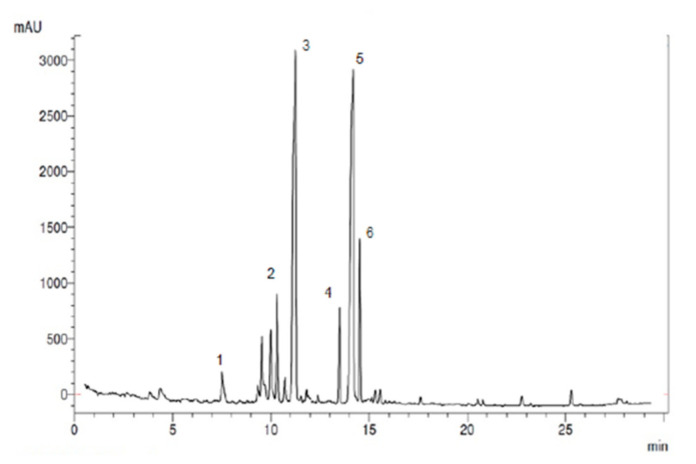
The typical HPLC-UV chromatogram of compositional polyphenols presented in HAW extract.

In the present study, the total antioxidant capacity (by the FRAP assay) and total polyphenolic content (by the Folin–Ciocalteu method) were calculated as ascorbic acid and gallic acid equivalent, respectively (Table 2). Each gram of dried HAW extract contains high total antioxidant capacity; equals to 877.8 µmol ± 5.33 ascorbic acid equivalent. We showed that each gram of dried HAW had a high total polyphenolic content; equal to 150.37 ± 1.33 mg gallic equivalent. Furthermore, the main polyphenols of HAW were identified and quantified by a validated HPLC-UV technique Figure 6 showed the typical HPLC-UV chromatograms of compositional polyphenols presented in HAW extract.

Six peaks were identified in the order of chlorogenic acid (7.2 min), vitexin (10.3 min), rutin (11.01 min), epicatechin (13.5 min), quercetin (14.01 min), and iso quercetin (14.5 min) (Figure 6) by comparisons to the retention times of standards analyzed under identical analytical conditions. The quantitative analysis was based on calibration curves obtained from the used standards. As Table 2 and Figure 6, HAW contains chlorogenic acid, vitexin, rutin epicatechin quercetin, and iso quercetin at the concentration of 5.22, 15.12, 30.77, 11.23, 32.14, and 17.05 mg/mg, respectively. The major compound present in the plant extract was identified as quercetin (32.14 mg/g) and rutin (30.77 mg/g) and the least abundant compound present in the extract was chlorogenic acid (5.22 mg/g).

## 4. Discussion

The current study was conducted to evaluate antifibrotic effect HAW treatment against CCl4-induced liver fibrosis. HAW not only decreased liver injury, and recovered liver function, but also reduced ECM deposition. These beneficial capacities are mediated at least in part through the reduction of HSC activation, the inhibition of inflammatory signaling pathway and the suppression of oxidative stress-trigged damage. As it mimics chronic liver diseases, CCl4 is a hepatotoxin that is commonly used to develop animal models of liver fibrosis when administered at low and repetitive doses. In liver tissue, CCl4 generates methyltrichloride radicals (CCl3·) that cause marked centrilobular liver necrosis, induction of the inflammatory response, activation of HSCs, and a build-up of ECM [8,33]. Similarly, in the present study, necrosis was accompanied by inflammation, hepatic stellate cells activation and fibrosis as was evident both by histological and biochemical analyses. In the present study, significant necrosis of liver cells and release of markers of liver injury such as AST, ALT, GGT, and T. bilirubin with a range of histological degenerative, inflammatory, and fibrotic reactions have all been induced in liver through repeated administration of CCl4. HAW remedy exerts hepatoprotective effects against hepatocellular damage shown in the fibrotic model developed in this study. This was evident through the attenuation of serum AST and ALT and the protection against hepatobiliary damage as reflected by the decrease of T. bilirubin level and of the GGT activity. These beneficial properties of HAW were also supported by the marked alleviation of fatty degeneration, necrosis and infiltration of inflammatory cells. HAW treatment not only prevented hepatic damage it also ameliorated the decline of liver synthetic function of serum albumin caused by chronic liver injury.

Development of liver fibrosis is driven by ongoing liver injury through multiple mechanisms where hepatic fibrosis is often caused by repeated liver damage whereby the everyday wound-healing response leads to a production and a buildup of ECM such as collagen [2,3]. Collagen 1 and 3 are the most important types of collagens, which can replace the basal membrane of the sub endothelial house of Disse area and therefore, if managed, may play a vital role in the improvement of liver fibrosis. Our study confirmed that CCl4 administration led to accumulation of collagen in the liver tissues as demonstrated through the result of the Masson’s trichrome staining and the increased level of HP; biochemical marker of collagen and the most representative amino acid present in collagen [35]. This was concomitant with the induced expressions of key hepatic proteins and mRNA (such as collagen-1 and -3). HAW treatment successfully alleviated all modifications of collagen brought about by CCl4 intoxication. These experimental results suggest that the HAW-mediated inhibition of ECM via blocking the synthesis and deposition of collagen may lay a strong pharmacological foundation of HAW as a novel anti-liver fibrosis drug. In fact, hepatic damage and fibrotic analyses showed a clear advantage of HAW over SIL as treatment against liver fibrosis.

Activation of HSCs is a central regulator responsible for the production of collagen and the progression of liver fibrosis [3,8]. Although, necrosis is one of the main causes of liver fibrosis induced by CCl4 administration, cell death by apoptosis can also induce fibrosis by activating HSCs which represent the core machinery to phagocytose apoptotic bodies [9,44]. Cellular changes accompanying HSC activation include morphological alterations of the cytoskeletal protein α-SMA [3,45]. This study showed that expressions of both the protein and mRNA levels of α-SMA were considerably induced in liver tissues of the CCl4 group. Therefore, the inhibitory effect on the activation of HSC may signify the most important target for anti-fibrotic agents. HAW inhibited hepatic fibrosis along with suppressing the α-SMA-producing cells in liver, suggesting the capacity of HAW to attenuate HSCs activation.

Inflammatory response and the release of inflammatory markers from damaged liver cells (hepatocytes and hepatic immune cells) are often a result of HSCs activation [5,32]. In the current work, evaluation of some inflammatory markers revealed that repeated administration of CCl4 induced hepatic expression of IL-1β, TNF-α, TGF β, COX-2 as well as NF-κB. Significant elevation in hepatic activity of the MPO as marker of neutrophils infiltrations indicated an extended inflammatory response throughout the duration of chronic hepatic injury. Since HAW substantially recovered the normal inflammatory microenvironment through inhibiting the expressions of pro-inflammatory factors, it actually manifested a potent anti-fibrotic effect. Our results agree with previous study that showed that HAW ingestion mitigated liver injury and inflammation induced by high fructose diet in mice by inhibiting release of inflammatory cytokines (TNF-α, IL-1, and IL-6) [31]. Other studies verified that the administration of HAW and CO extract treatment reduced inflammatory cytokine (TNF-a, IL-1, and IL-6) [31] and MPO activity [25] in different animal models.

In addition, excessive ROS accumulation is a fundamental driver of hepatic inflammation and fibrosis [46]. Excessive ROS induced through CCl4 intermediates, may act as signaling messenger to NF-kB, that eventually lead to elevated cytokine production, including COX-2, IL-6, and TNF-a and inflammation [8,32]. In the current study, oxidative damage was observed in liver of CCl4-treated rats as confirmed by induced lipid peroxidation (MDA), and protein oxidation (P.Carbonyl) and by the reduction of SOD activity in liver. On the other hand, HAW administration effectively decreased the CCl4-induced oxidative stress as evident through preventing the elevation in MDA and P.Carbonyl levels and ameliorating the reduction of SOD activity in liver. In the present model of chronic liver damage, HAW decreased the release of damage-associated reactive oxygen species and other fibro genic mediators and recruitment of inflammatory cells, which in turn mediate HSC activation and stimulate collagen secretion through release of cytokines and chemokines. This shows that antioxidant property of HAW may be the main mechanism involved in hindering CCl4-induced toxicity and fibrosis. The administration of extracts from CO and from other species have been reported to increase antioxidant capacity, the expression of SOD, and catalase, with the subsequent decline of MDA in animal model of non-alcoholic fatty liver ailment [27,31] and myocardial injury [24,25]. Thus, the protective effect HAW could be attributed to the activation of endogenous antioxidants such as SOD and CAT that counteract the oxidative damage which is induced by ROS. Similarly, we observed a prominent association between the antioxidant activity and the phenolic contents in of HAW extract where phenolic compounds seem to be accountable for the antioxidant activity of HAW extract. In this context, previous studies confirmed that the HAW leaves and flowers from CO species contained a high content of phenolic compounds that was correlated with excessive antioxidant capacity. Previous investigations on CO [18,21,47] showed that flower and leaves of CO contained phenolic compounds like rutin, quercitrin chlorogenic acid, hyperoside, vitexin, and caffeic acid that represent the most compounds. Here, we too showed that HAW extracts contained high total phenolic content. The principle phenolic compounds found in CO extract include chlorogenic acid, epicatechin, rutin, vitexin, quercetin, and iso quercetin used to be the principal compound in which is constant with the previous studies [18,21,47]. Throughout the course of this study HAW at dose 350 mg/kg did not induce any sign of toxicity in rats. Similarly, no signs of toxicity have been observed after oral application of doses up to 3000 mg/kg and an LD50 of 1750 mg/kg for intraperitoneal administration was described in animal studies [23,48]. Despite this, further experiments are needed to confirm the dose-dependent efficacy and safety of another animal model.

## 5. Conclusions

**Figure 7:**
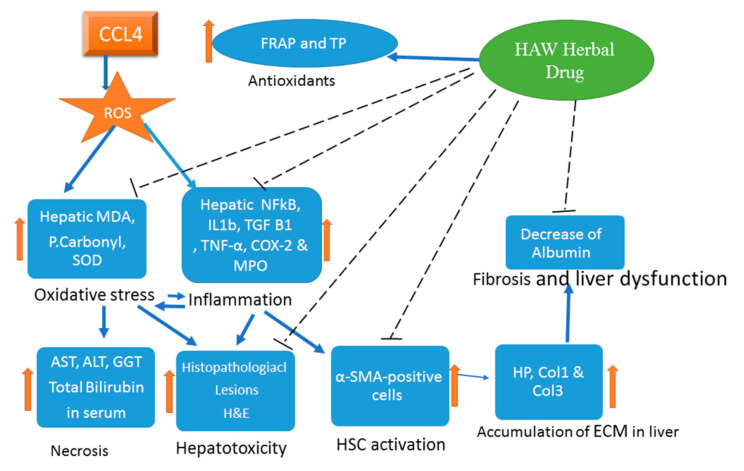
A graphical abstract highlighting the protective effect of HAW in CCl4-induced liver fibrosis by blocking the pro-oxidant machinery and the downregulation of the inflammatory and profibrotic responses. The protective effects include reducing deposition. Liver toxicity, improving the liver histology and decreasing collagen.

The present study, HAW seems to restore normal liver characteristics of animals induced for liver fibrosis. We investigated here the crosstalk between liver damage, oxidative stress, inflammation, and activation of fibrogenesis. HAW appears to function via modulation of oxidative stress, reduction of inflammation and inhibition of HSC activation (Figure 7). Accordingly, the present findings introduce HAW as a novel drug candidate for treating liver fibrosis. However, these observations require additional experiments for verification. Further studies are expected to elucidate the pharmacodynamics and pharmacokinetics of this herbal medication. In addition, whether HAW’s beneficial effects can be manifested in humans awaits future clinical trials.

## Figures and Tables

**Table 1:** Primer sequences used for reverse-transcriptase-polymerase chain reaction (RT-PCR).

Gene Name	Primer Sequence: 5′-3′	Gene Bank Accession Number
TNF-α	F: AACTCGAGTGACAAGCCCGTAG R: GTACCACCAGTTGGTTGTCTTTGA	NM_012675.3
NF-kB	F: CATTGAGGTGTATTTCACGG R: GAACACAATGGCCACTTGCC	NM_199267.2
IL-1β	F: GCTGTGGCAGCTACCTATGTCTTG R: AGGTCGTCATCATCCCACGAG	NM_031512.2
COX-2	F: ACTTGCTCACTTTGTTTCATTC R: TTTGATTAGTACTGTAGGGTTAATG	S67722.1
TGF-β	F: TGCGCCTGCAGAGATTCAAG R: AGGTAACGCCAGGAATTGTTGCTA	NM_021578.2
α-SMA	F: ACCAACTGGGACGACATGGAG R: CGTGAGGATCTTCATGAGGTAGTC	NM_031004.2
Colla1	F: GAACTTGGGGCAAGACAGTCA R: GTCACGTTCAGTTGGTCAA	NM_053304.1
Colla3	F: TTGATGTGCAGCTGGCATTC R: GCCACTGGCCTGATCCATAT	NM_009930
GAPDH	F: CCCCTTCATTGACCTCAACTACATGG R: GCCTGCTTCACCACCTTCTTGATGTC	NM_017008.4

**Table 2:** Amount of total antioxidant ^a^ phenolics ^b^, and the qualitative–quantitative analyses of phenolic compounds of HAW extract carried out using an HPLC.

	Compound	Retention Time/min	Amount of Compounds
a	Total antioxidants content	-	877.8 ± 5.33 µmol/g
b	Total phenolic content	-	150.37 ± 1.33 mg/g
1	Chlorogenic	7.2	5.22 ± 0.01 mg/g
2	Vitexin	10.3	15.12± 0.13 mg/g
3	Rutin	11.2	30.77± 0.13 mg/g
4	Epicatechin	13.5	11.23 ± 0.01 mg/g
5	Quercetin	14.01	32.14 ± 0.11 mg/g
6	Iso quercetin	14.5	17.05 ± 1.13 mg/g

Results are expressed as mean ± SEM. ^a^ Total antioxidant activity of ethanol extract from HAW expressed as ascorbic acid equivalents (µmol/g of dry extract). ^b^ Total phenolic content expressed as mg gallic acid equivalents/g dried. Extract.1-6 the amounts of compounds expressed as mg/g of dried extract.

## Abbreviations

HAW	Hawthorn
CCl4	Carbon tetrachloride
CO	Crataegus oxyacanthine
IL1B	interleukin-IL-1β
TNF-α	tumor necrosis factor-α
TGF-β 1	transforming growth factor-β 1
NFkB	nuclear factor kappa-B
COX-2	cyclooxygenase-2
MPO	the myeloperoxidase
MDA	malondialdehyde
P.Carbonyl	Protein carbonyl
SOD	superoxide dismutase
α-SMA	alpha-smooth muscle actin
SIL	silymarin
ROS	reactive oxygen species
HSC	hepatic stellate cells
H & E	hematoxylin and eosin
HP	hydroxyproline
TAC	total antioxidant capacity
FRAP	ferric reducing antioxidant power
AST	aspartate aminotransferase
ALT	alanine aminotransferase
GGT	gamma-glutamyl transferase
